# Labrador Sea freshening linked to Beaufort Gyre freshwater release

**DOI:** 10.1038/s41467-021-21470-3

**Published:** 2021-02-23

**Authors:** Jiaxu Zhang, Wilbert Weijer, Michael Steele, Wei Cheng, Tarun Verma, Milena Veneziani

**Affiliations:** 1 Computational Physics and Methods, Los Alamos National Laboratory, Los Alamos, NM USA; 2 Center for Nonlinear Studies, Los Alamos National Laboratory, Los Alamos, NM USA; 3 Joint Institute for the Study of the Atmosphere and Ocean, now Cooperative Institute for Climate, Ocean, and Ecosystem Studies, University of Washington, Seattle, WA USA; 4 NOAA/Pacific Marine Environmental Laboratory, Seattle, WA USA; 5 Applied Physics Laboratory, University of Washington, Seattle, WA USA; 6 Fluid Dynamics and Solid Mechanics, Los Alamos National Laboratory, Los Alamos, NM USA

**Keywords:** Climate and Earth system modelling, Physical oceanography, Physical oceanography

## Abstract

The Beaufort Gyre (BG), the largest Arctic Ocean freshwater reservoir, has drastically increased its liquid freshwater content by 40% in the past two decades. If released within a short period, the excess freshwater could potentially impact the large-scale ocean circulation by freshening the upper subpolar North Atlantic. Here, we track BG-sourced freshwater using passive tracers in a global ocean sea-ice model and show that this freshwater exited the Arctic mostly through the Canadian Arctic Archipelago, rather than Fram Strait, during an historical release event in 1983–1995. The Labrador Sea is the most affected region in the subpolar North Atlantic, with a freshening of 0.2 psu on the western shelves and 0.4 psu in the Labrador Current. Given that the present BG freshwater content anomaly is twice the historical analog studied here, the impact of a future rapid release on Labrador Sea salinity could be significant, easily exceeding similar fluxes from Greenland meltwater.

## Abstract (web summary)

The Beaufort Gyre in the western Arctic Ocean has accumulated a large amount of freshwater. Here, the authors show that a historical release in the 1980s resulted in a strong freshening of the western Labrador Sea, suggesting that a future release of the current freshwater volume could even be more impactful.

## Introduction

The anticyclonic Beaufort Gyre (BG) in the western Arctic Ocean maintains the largest oceanic freshwater reservoir in the northern hemisphere^1^. Observations^2^ indicate that its liquid freshwater content has reached a record high value of 23,300 km^3^ in 2017, a result of several factors, including an anomalously persistent anticyclonic (clockwise) wind regime over the BG region and sea-ice decline associated with warming of the Arctic^2–4^. If released via eddies or gyre spin-down^5,6^, this high volume of freshwater anomaly may be transported to the subpolar North Atlantic and decrease its upper ocean salinity and density. Given that the subpolar North Atlantic is a site of deep-water formation, such freshening could have consequences for the strength of the Atlantic Meridional Overturning Circulation (AMOC) and its global impacts^7,8^.

However, our understanding of the downstream impacts of the BG freshwater is limited by the absence of reliable estimates of the exact pathways of freshwater flow from the BG to the North Atlantic, and the magnitude of salinity anomalies induced by such a release. While existing studies have focused on the sources of freshwater to the BG^2,9,10^ and causes of its recent drastic accumulation^2,11^, quantitative studies focusing on the fate of freshwater after it leaves the BG are relatively uncommon. Specifically, the pathway of freshwater release from the BG through either the straits of the Canadian Arctic Archipelago (CAA, also known as the Canadian Polar Shelf) toward the Labrador Sea or through Fram Strait toward the Nordic Seas has rarely been explored. Moreover, while observational and modeling studies have examined the overall pan-Arctic freshwater budget as well as its impact on the North Atlantic salinity^12–17^, no studies have specifically differentiated the role of the BG region from the rest of the Arctic Ocean. For example, modeling studies^18,19^ have shown that Arctic freshwater can be released by forcing the Arctic Ocean with idealized cyclonic wind conditions, but they were not able to identify how much of the release is contributed by water sourced from the BG region.

The impact of BG freshwater on North Atlantic salinity is difficult to assess in numerical models for several reasons. Low-resolution models (typically 1^∘^) cannot resolve the complex channel network of the CAA and usually simplify the region to one or two large straits^9,15,16^ or assume its transport to be small^20,21^. Regional high-resolution models, varying from 1/4^∘^ to 1/12^∘^, feature much better representation of the CAA but usually lack the geographic coverage required to explore the impact of Arctic–Atlantic freshwater exchanges on ocean salinities at lower latitudes^18,19,22^. Global high-resolution models are generally computationally too expensive to be used for long-term simulations. Finally, a technically challenging question is how to quantitatively estimate the salinity anomalies induced specifically by BG freshwater. Such a quantification cannot be achieved by traditional model diagnostics (e.g., volume transport, freshwater transport), Lagrangian tracer particles^11,23,24^, or passive dye tracers^9,10,25^ alone.

**Fig. 1:**
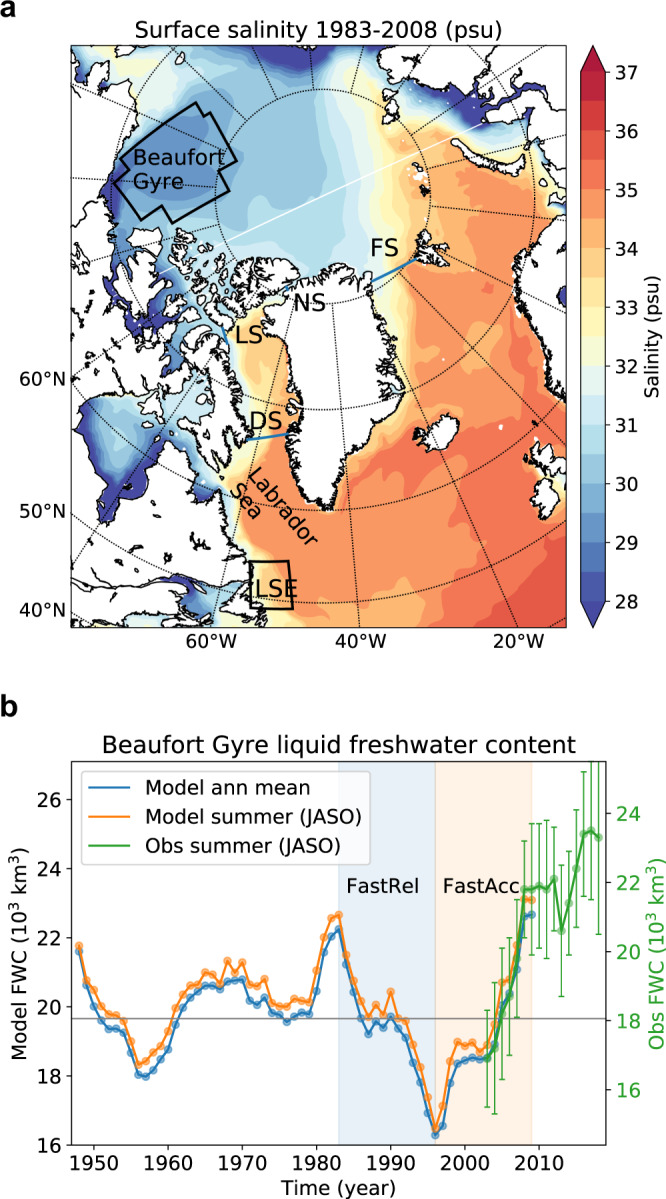
Study region showing the Beaufort Gyre (BG) and major ocean gateways, as well as the BG freshwater content. **a** Climatological surface salinity in the Arctic and the North Atlantic in our HiLAT03 ocean sea-ice model for 1983-2008. Black zigzag box indicates the BG region. Black box exit of Labrador Sea (LSE), NS Nares Strait, LS Lancaster Sound, DS Davis Strait, FS Fram Strait. **b** Annual mean (blue) BG liquid freshwater content relative to 34.6 psu, which is the modeled mean salinity of the Arctic Basin (Supplementary Note 1). Its mean value of 19,660 km^3^ is indicated by the horizontal gray line. The summer average (July–October; orange) is also shown to be compared with the 2003–2018 observation^64^ (green with error bars indicating root-mean-square error of freshwater content interpolation; an offset of 1600 km^3^). FastRel and FastAcc are tracer experiments for 1983–1995 and 1996–2008, respectively.

Here, we aim to quantify the impacts of a BG freshwater release by comparing transport pathways and downstream freshening during two historical episodes of rapid BG freshwater release and accumulation, using an eddy-permitting (1/3^∘^) ocean-sea ice model^26^. Our passive tracer design (see Methods section) tags both volume and salinity within the BG freshwater lens, that is, from the surface to the depth of the reference salinity within the BG region (black box in Fig. 1a). This method allows an assessment of both the volume and equivalent freshwater transport of BG-sourced water as well as the salinity changes it induces at any downstream location without introducing any artificial perturbations into the system. The tracers track only the liquid freshwater of the BG and not the solid freshwater in the ice, although the latter also plays a role in the oceanic freshwater cycle^3,27^. Our results show that historical BG-sourced freshwater enters the North Atlantic mostly through the CAA and freshens the Labrador Sea western shelves. This BG-originated freshening may have contributed to the surface freshening observed in the early 1990s known as the Great Salinity Anomaly 90s^28,29^, and also is comparable to the impact associated with present and near-future Greenland meltwater input^30,31^.

## Results

### Experimental design

We first perform a hindcast simulation using prescribed atmospheric forcing over the period 1948–2009^32^. The model produces realistic volume and freshwater fluxes through major Arctic gateways (Supplementary Table 1), while the main characteristics of the stratification in the Arctic Ocean are adequately represented in the model (Supplementary Note 1) –despite some obvious and common biases^33^. The slight fresh bias is corrected for by choosing a lower reference salinity (34.6) than is commonly used (34.8) for defining freshwater^34^. The simulated BG freshwater content shows a pronounced decadal variability, and more importantly, reproduces the observed^2^ rapid increase of BG freshwater content during 2003–2009 (Fig. 1b).

Then we select two periods to tag BG water with passive tracers: 1983–1995 corresponding to a period of rapid release of freshwater from the BG (our fast-release case, or FastRel), and 1996–2008 corresponding to a period of rapid freshwater accumulation in the BG (our fast-accumulation case, or FastAcc; Fig. 1b). The passive tracers are for diagnosis only and do not feed back on the model dynamics. The BG region releases a net amount of 6000 km^3^ freshwater during the 13-year FastRel period and accumulates roughly the same amount in the 13-year FastAcc period. Differences between these two scenarios (FastRel minus FastAcc) are used to diagnose the preferred transport routes and associated salinity anomalies in the North Atlantic Ocean. During all years, water with fresh signature leaves the BG and flows into the North Atlantic Ocean, owing to both local wind and other forcings as well as the large-scale sea level height gradient^35^. However, during the FastAcc episode, the amount of freshwater that leaves the BG is strongly reduced compared to the FastRel episode.

### Changes in the Arctic Basin

**Fig. 2:**
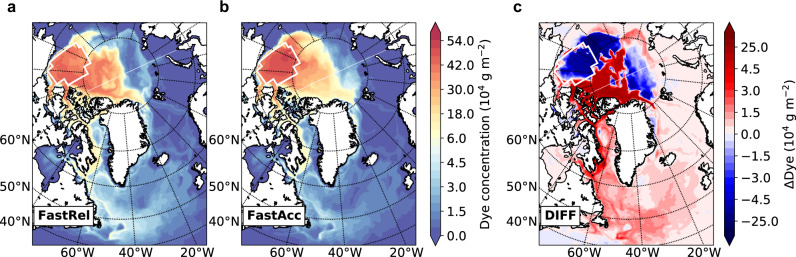
Fate of the Beaufort Gyre (BG) freshwater in different scenarios. Distribution of vertically integrated dye tracer concentration by the end of each 13-year simulations of FastRel (1983–1995 with rapid release of freshwater from the BG; **a**), FastAcc (1996–2008 with rapid freshwater accumulation in the BG; **b**), and the difference (FastRel minus FastAcc) between them (**c**). White boxes indicate the BG region where tracers are released.

Our tracer experiments reveal significant differences between the FastRel and FastAcc episodes, illustrated by the dye tracer concentrations tracking the fate of BG-sourced water (Fig. 2). Both episodes show highest concentrations in the Western Arctic within the Canada and Makarov basins and two distinct release pathways into the Atlantic: one through the CAA and then Baffin Bay and Davis Strait, and the other through Fram Strait. However, within the Arctic Basin, FastRel tends to transport freshwater directly toward the CAA and north of Greenland, while FastAcc tends to accumulate freshwater locally within the BG, while also transporting it into deeper BG layers and also toward the East Siberian Sea (Fig. 2c). This reflects differences in the ocean circulation between the two periods: during the FastRel period, the BG spins down due to an anomalously cyclonic wind stress regime^2^, and the Atlantic/Pacific front rotates cyclonically from its long-term mean position (Supplementary Fig. 1). This allows significant outflow of the BG freshwater toward the CAA and north of Greenland^36^. In contrast, during the FastAcc period, the BG spins up and the front rotates in the opposite direction, promoting local accumulation and westward transport. As a result, more freshwater enters Baffin Bay, the Labrador Sea, and the subpolar gyre in FastRel than in FastAcc, the details of which are quantified below.

### Transports through Arctic gateways

**Fig. 3:**
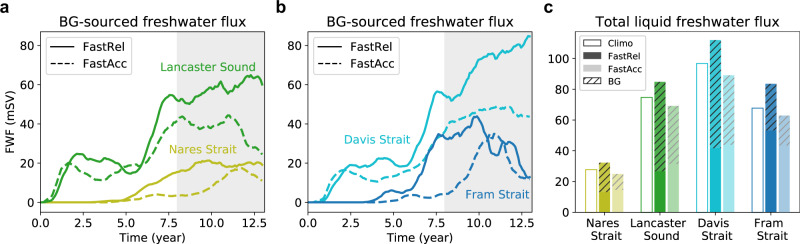
Beaufort Gyre-sourced freshwater flux at major Arctic–Atlantic ocean gateways. Time series of the Beaufort Gyre-sourced freshwater flux during FastRel (solid) and FastAcc (dashed) periods at two Canadian Arctic Archipelago pathways, Lancaster Sound and Nares Strait (**a**), as well as Davis and Fram Straits (**b**). Locations of these gateways are shown in Fig. 1a. Lines are 12-point unweighted running averages based on monthly output. **c** Total liquid freshwater flux averaged over the last 5 years (gray patches in **a** and **b**) of the FastRel (dark) and FastAcc (light) periods, compared with the long-term climatologies (unfilled boxes). Hatched areas of the bars indicate the Beaufort Gyre-sourced contributions.

Quantitative estimates of the liquid freshwater flux via major Arctic/Atlantic ocean gateways indicate that the main release route of BG water into the North Atlantic is through Davis Strait rather than through Fram Strait (Fig. 3). Davis Strait collects transport through two major CAA outflow routes: Parry Channel with Lancaster Sound at the end, and Nares Strait (locations shown in Fig. 1a). Climatological freshwater flux through Lancaster Sound simulated in the model is 74.7 mSv (1 mSv = 10^3^ m^3^s^−1^), nearly three times greater than that through Nares Strait (Fig. 3c), although the volume fluxes at the two gateways are more comparable (0.96 Sv vs. 0.69 Sv; Supplementary Table 2; see Methods section). It takes only a few months for the tracers to arrive at Lancaster Sound, since Parry Channel is directly connected to the BG. In contrast, it takes about 5 years before a significant amount of tracer can be detected at Nares Strait, owing to the longer, slower pathway from the BG via the Transpolar Drift Stream^36^ (Fig. 3a). At Davis Strait, BG-sourced freshwater flux is 70 mSv in FastRel, compared to 45  mSv in FastAcc, averaged over the last 5 years of each period (Supplementary Table 2). At these three straits (Lancaster Sound, Nares Strait, and Davis Strait), BG-sourced water dominates the total liquid freshwater flux (the exception being that BG-sourced water accounts for 41% at Nares Strait in FastAcc; Fig. 3c). Also, the contribution from non-BG waters to the freshwater fluxes through these straits is largely the same for the FastRel and FastAcc periods, suggesting that the differences in net freshwater flux can be directly attributed to BG changes. In contrast, BG-sourced water is not the dominant component of liquid freshwater flux through Fram Strait, where it accounts for only 38.2% in FastRel and 31.8% in FastAcc.

### Impact on downstream salinity

**Fig. 4:**
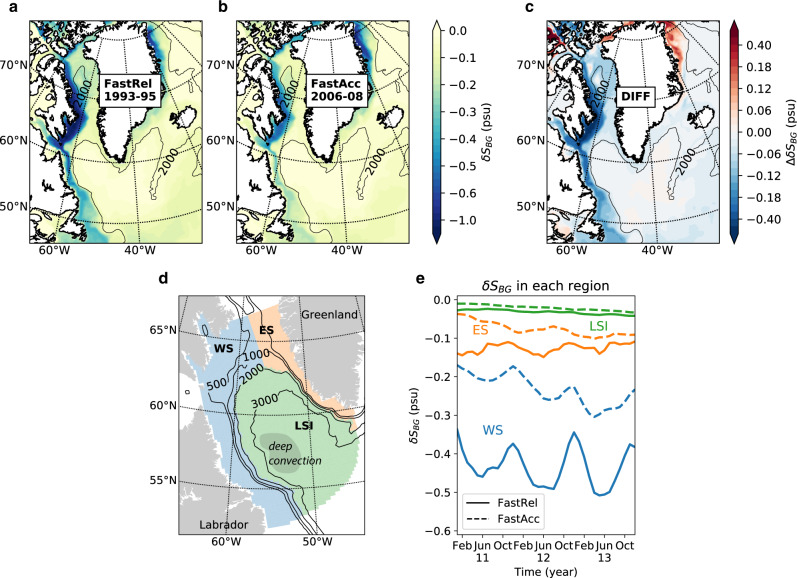
Impact on downstream salinities. Changes of upper-200-m salinity induced by the Beaufort Gyre-sourced water during the last 3 years of FastRel (**a**), FastAcc (**b**), and the differences (FastRel minus FastAcc) (**c**). Beaufort Gyre-induced salinity changes (*δS*_BG_) are estimated as the difference between the local salinity and the salinity of the non-Beaufort Gyre-sourced water, averaged over the upper 200 m (see Methods section). The 2000 m isobath is shown in thin black lines. **d** The Labrador region is divided into western shelves (WS), eastern shelves (ES), and Labrador Sea interior (LSI), as shown by colors. The 2000 m isobath is used to separate the basin interior from the shallower regions. Gray shading in the LSI schemes the main deep convection region as observed. **e** Monthly *δS*_BG_ averaged over the three sub-regions as indicated in **d**.

Our tracer diagnoses reveal a profound influence of BG freshwater on the salinity of downstream regions. In particular, during the last 3 years of FastRel (1993–95), BG freshwater lowers the upper-200-m salinity (*δS*_BG_, in psu) by up to 1.2 in western Baffin Bay and 0.6 in the western Labrador Sea (Fig. 4a). There is also a smaller impact in the East Greenland Current. This estimation is based on a method that makes use of the dye tracer and salt tracer and accounts for the impact from BG-sourced water alone (see Methods section). The corresponding salinity changes in the FastAcc scenario (2006–2008) show similar patterns, but with approximately half of the magnitude over western Baffin Bay and the western Labrador Sea (Fig. 4b, c). Interestingly, the BG-induced freshening is weaker in FastRel than in FastAcc in Nares Strait and the northern East Greenland Current (red areas in Fig. 4c). The reason is that during the FastRel case, Nares and Fram straits are clearly under the influence of fresher Pacific waters (not from the BG), due to the cyclonic rotation of the Pacific/Atlantic front (Supplementary Fig. 1). This means that BG-induced freshening is weaker in these areas during FastRel because the base state (i.e., without BG influence) is already fresh owing to Pacific water influence. In contrast, this area is influenced by saltier Atlantic waters during the FastAcc case, and so BG freshwater influence is stronger. Another factor is that BG-sourced water enters deeper layers in these areas during FastRel (Supplementary Fig. 2), leaving the shallow coastal regions affected more by non-BG Pacific water sources (Supplementary Fig. 3).

We now focus on impacts of BG-sourced freshwater in the Labrador Sea, an important deep-water formation region of the North Atlantic. The western shelves are the most affected area (blue areas in Fig. 4d), where BG freshwater is responsible for a salinity change of −0.43 averaged over 1993–1995; this is almost twice the −0.23 change averaged over 2006–2008 (Table 1). Further examination of the time series indicates that the BG-induced freshening continues through the final 3 years of the simulations (Fig. 4e). The maximum impacts are usually found in late summer, when the water column is maximally stratified and thus keeps freshwater close to the surface (Fig. 4e). In contrast, the BG impact over the eastern shelves and Labrador Sea interior is much smaller and similar between the two periods (see Table 1 for numbers). We also note that although a BG impact in the upper layers of the basin interior is hardly seen, there are non-negligible full-column tracer concentrations in this region (Fig. 2). This suggests that much of the tracer has been transported below 200 m (Supplementary Fig. 4). Our tracer analysis implies that, compared to FastAcc, BG-sourced freshwater during the FastRel period reduces the upper 200 m salinity of the Labrador Sea western shelves by 0.2, while locally along the Labrador Current salinity is reduced by 0.4 (Fig. 4c). This freshening is associated with an *O*(25 mSv) increase in the BG-sourced freshwater flux through Davis Strait (difference between FastRel and FastAcc; Supplementary Table 2), and an anomalous freshwater transport of 5600 km^3^, when integrated over the full 13 years.

**Table 1:** Labrador Sea freshening induced by Beaufort Gyre freshwater.

		Surface				Upper 200 m			
Region	Episode	*S*	*S*_nBG_	*δS*_BG_	Δ*δS*_BG_	*S*	*S*_nBG_	*δS*_BG_	Δ*δS*_BG_
Western shelves	FastRel	32.81	33.07	−0.27	**−0.12**	33.58	34.02	−0.43	**−0.20**
	FastAcc	33.13	33.28	−0.15		33.81	34.05	−0.23	
Eastern shelves	FastRel	34.45	34.57	−0.11	**−0.04**	34.58	34.71	−0.13	**−0.05**
	FastAcc	34.72	34.79	−0.07		34.80	34.88	−0.08	
Labrador Sea interior	FastRel	34.82	34.85	−0.03	**−0.01**	34.88	34.91	−0.03	**−0.01**
	FastAcc	34.89	34.91	−0.02		34.96	34.98	−0.02	

Changes in surface and upper-200-m salinities induced by BG-sourced water (*δS*_BG_) at the western shelves, eastern shelves, and interior Labrador Sea regions (Fig. 4d) calculated for the last three years of the FastRel and FastAcc episodes. *δS*_BG_ is estimated as the difference between the local salinity (*S*) and the salinity of the non-BG-sourced water (*S*_nBG_). Δ*δS*_BG_ indicates the difference in *δS*_BG_ between the two cases (FastRel minus FastAcc) and is shown in bold numbers. See Methods section for details. All symbols used here are consistent with those in Fig. 4.

### Comparison with Greenland meltwater flux

To put these BG-induced salinity changes into context, we compare them with those induced by Greenland meltwater flux as estimated by other modeling studies. Those studies are motivated by a rapid increase of Greenland meltwater flux of 12 mSv from 1996 to 2013 documented by satellites; a 50% increase relative to its climatological value of 24 mSv^37,38^. One study^31^ forced an eddy-resolving model with a linearly increasing trend of Greenland meltwater flux for 30 years, corresponding to a flux anomaly of 16.4 mSv by 2019 and a cumulative runoff anomaly of 7500 km^3^. This model produced a sea surface salinity (SSS) change of −0.3 along the West Greenland Current and −0.1 over the interior Labrador Sea. By introducing an averaged Greenland meltwater flux of 9 mSv during 2008–2012, another eddy-resolving model^30^ estimated a maximum SSS change of −0.32 over the interior Labrador Sea. These numbers can be compared with our results, where a BG-sourced freshwater flux anomaly of *O*(25 mSv; or an anomalous transport of 5600 km^3^ over a 13-year period) would induce an upper-200-m salinity anomaly of up to −0.4 along the Labrador Current and −0.2 over the western shelves. The equivalent BG-induced SSS anomalies are slightly smaller (Table 1 and Supplementary Note 2). Thus, SSS anomalies induced by anomalous BG fluxes and Greenland ice melt are of comparable magnitudes, although they impact different locations within the Labrador Sea.

## Discussion

We have shown that the CAA and the downstream Davis Strait, rather than Fram Strait, are the main pathways through which BG freshwater exits the Arctic Ocean into the North Atlantic Ocean (Fig. 3). Previous modeling studies have found an increase of liquid freshwater transport through the CAA when forcing the Arctic Ocean with idealized, large-scale, and cyclonic winds^18,19^; our results of the freshwater decomposition (Fig. 3c) further suggest that the increased CAA freshwater transport is dominated by water from the BG region as compared to other regions of the Arctic.

We have shown that an anomalous BG freshwater flux of *O*(25 mSv) through Davis Strait induces a freshening of 0.2 over the Labrador Sea western shelves. A key follow-up question is whether the BG-induced salinity changes affect deep-water formation over the Labrador Sea and to what extent. In order to discuss this question, we take three elements into consideration. The first is winter atmospheric conditions over the Labrador Sea. Comparing modeled upper-layer salinity with observations taken at the exit of the Labrador Sea near 51^∘^N (Fig. 1a and Supplementary Fig. 5), we find that the model is able to reproduce the observed freshening trend during the Great Salinity Anomaly 90s^28,29^, and the subsequent salinification during the 2000s. Our analysis shows that the main cause of such salinity changes over the Labrador Sea western shelves is the BG-sourced water, with non-BG-sourced water largely unchanged (Table 1). However, observations^39^ suggest that this freshening did not reduce the deep-water formation rate over the Labrador Sea as expected; instead, a record convection and strong formation of Labrador Sea Water were observed during the early 1990s, influenced by several harsh winters associated with repeated positive phases of the winter North Atlantic Oscillation^39^. This is in contrast to a previous GSA event during 1970s, which had stronger freshening, encountered mild winters and led to cessation of deep-water formation^40^. Thus, our results suggest that BG-sourced freshwater is able to induce GSA-magnitude salinity anomalies over the western shelves, but whether such salinity anomalies are able to significantly affect the deep-water formation rate depends strongly on winter atmospheric conditions.

The second element to consider is shelf-basin exchange induced by mesoscale eddy activity, which transports freshwater from the coastal boundary current into the Labrador Sea interior^31,41,42^. However, the model resolution required to accurately simulate such a process is still debatable^43^. Eddy parameterizations in low-resolution models (⩾1^∘^) tend to overestimate this eddy transport, hence effectively suppressing convection^9,16,17^. As resolution increases through the eddy-permitting regime from 1/3^∘^ (22 km)^41,44^ to 1/6^∘^ (18 km)^18^ and 1/8^∘^ (13 km)^19^, models start to resolve the narrow boundary currents, but cross-shelf transport may still be underestimated. Only when eddies are explicitly resolved by model grids finer than, say, 1/10^∘^ (9 km) can shelf-basin exchange be expected to actively transport boundary freshwater to convection-active sites^22,31^. In our model of 1/3^∘^ (20 km over this region), we have adopted a flux-limited tracer advection scheme^45^ and turned off explicit eddy parameterization to optimize eddy activity in the model and properly resolve boundary currents^26^. The resulting eddy activity in the simulation measured by sea surface height variability is comparable to observations^26^. Nonetheless, it is fair to assume that in our model freshwater transport towards the interior of the Labrador Sea is somewhat suppressed compared to what can be achieved by models with higher resolution.

Although we have discussed deep convection in the Labrador Sea interior, whether such deep convection directly results in deep-water formation and links to AMOC variability is a third element to be considered. The fact that mesoscale eddy activity is stronger over the eastern side of Labrador Sea than the west^46–48^ may imply that Greenland meltwater can access the central basin and impact deep-water formation more easily than BG-sourced water can from the western shelves^31,41^, based on a common assumption that Labrador Sea Water formed in the central basin is directly linked to AMOC variability^49,50^. However, some recent observational and modeling studies have raised questions about such a link^51–53^. Instead, they suggest it is the buoyancy fluxes (from summer freshwater input or winter cooling) over steep topography such as the Labrador Sea continental slope (Fig. 4a) that primarily contribute to downward mass flux and ultimately the AMOC^54,55^. Both BG-sourced water and Greenland meltwater generally are found above steep continental slopes of the Labrador Sea (although on opposite sides), and in this regard, they both have the potential to perturb the AMOC. Our focus on the western Labrador Sea salinity anomaly is partially motivated by this consideration.

In summary, we have quantified salinity change in the high-latitude North Atlantic induced by freshwater flux anomalies from the BG, using a global eddy-permitting ocean-sea ice model with realistically resolved CAA and a unique tracer design. We find that the CAA is an effective conduit that connects BG freshwater and the high-latitude North Atlantic; specifically, we find that an anomalous *O*(25 mSv) BG-sourced freshwater flux (through CAA then Davis Strait), corresponding to an anomalous transport of 5600  km^3^ over a 13-year period, may have lowered the upper-layer salinity by 0.2 on the Labrador Sea western shelves and 0.4 in the Labrador Current. In 2017, BG freshwater content reached an unprecedented positive anomaly, doubling the magnitude of the historical maximum in 1983 (Fig. 1b). If released within a short period similar to the FastRel episode and at a similar speed, it is capable of imposing a significant freshening over the western shelves of the Labrador Sea that would exceed freshening induced by, for instance, accelerated Greenland ice sheet and Arctic sea ice melting^56^. In fact, such a process may have already started^57^.

## Methods

### Model and simulation

The model used in this study is an eddy-permitting, global ocean-sea ice configuration^26^ of the E3SMv0-HiLAT (Energy Exascale Earth System Model version 0 configured for High-Latitude Application and Testing) model^58^ developed at Los Alamos National Laboratory. This model configuration, which we refer to as HiLAT03, has been documented in Zhang et al.^26^. Here we provide a brief summary.

The ocean component uses Parallel Ocean Program version 2^45^, which has a horizontal grid with nominal resolution of 1/3^∘^ ranging from 33 km in the tropics to 8.5 km at high latitudes. It has 100 vertical levels, ranging in thickness from 6 m at the surface to 150 m at depth, and uses partial bottom cells to represent bathymetry accurately. It uses the flux-limited Lax-Wendroff advection scheme and KPP for vertical mixing; there is no explicit parameterization for horizontal diffusion. Sea surface salinity is restored to the World Ocean Atlas 2013 version 2 (WOA13v2) climatology^59,60^ with a restoring time scale of 4 years over the open ocean (i.e., when there is no sea ice in a model grid cell), to prevent the model from drifting too far from climatology. The sea-ice component uses CICE5^61^, and runs on the same horizontal grid as the ocean model. CICE5 solves dynamic and thermodynamic equations for multiple ice thickness categories in each horizontal grid cell.

The HiLAT03 model was initialized from the Polar Science Center Hydrographic Climatology (PHC2) temperature and salinity data set^62^ and was forced by an inter-annually varying data set of the Coordinated Ocean-Ice Reference Experiments version 2 (CORE-II^32^) from 1948 to 2009 for three cycles (186 model years in total). The third forcing cycle is used for the tracer experiments and the analyses done in this study.

### Tracer design

Two types of passive Eulerian (scalar) tracers were implemented in the model to track the downstream transport of BG freshwater. The first type is a commonly used dye tracer (*α*_BG_), which is initialized as zero globally except in the BG domain (Fig. 1a) for layers fresher than *S*_ref_ (34.6 psu in our case), where it is kept as 1 g kg^−1^ at each time step. We use a zigzag BG region to maximize its overlap with the traditional definition^63^ (70.5^∘^N to 80.5^∘^N and 130^∘^W to 170^∘^W with water column >300 m) while keeping the boundaries aligned with the model grid to allow easy tracer release (Supplementary Fig. 6). In this way, the time-varying BG freshwater ‘lens’ (or ‘bowl’ in some other references) is dyed. Outside of the BG freshwater bowl, the dye tracer is passively transported by the advection and diffusion schemes and does not feed back to the ocean dynamics. The dye tracer represents the volume percentage of BG-sourced water in each grid cell.

The second type is a novel salt tracer (*D*_BG_), which shares a similar definition to the dye tracer except that it is reset to the local salinity instead of 1 g kg^−1^ within the BG freshwater bowl. Conceptually, *D*_BG_ = *α*_BG_⋅*S*_BG_, that is, the salt tracer can be thought of as the product of the volume percentage of the BG-sourced water and its salinity at the source, *S*_BG_. As explained below, this tracer allows us to diagnose the BG-sourced freshwater transport. Note that the restoring treatment of the surface salinity is not applied to any of the tracers, and the impact on our analysis is discussed in Supplementary Note 2.

The total volume transport (VT) and the BG-sourced volume transport (VT_BG_) across any downstream section (e.g., Nares Strait) are calculated as:

1 $${\mathrm{VT}}={\int}_{\!\!\!\!\!x}{\int}_{\!\!\!\!\!z}v\,{\mathrm{d}}z{\mathrm{d}}x,\quad {\mathrm{V{T}}}_{{\rm{BG}}}={\int}_{\!\!\!\!\!x}{\int}_{\!\!\!\!\!z}{\alpha }_{{\rm{BG}}}v\,{\mathrm{d}}z{\mathrm{d}}x,$$

where *v* is flow velocity perpendicular to the section, and integration is done across the section horizontally and vertically. The total freshwater transport (FWT) and BG-sourced freshwater transport (FWT_BG_) are calculated as:

2 $${\mathrm{FWT}}={\int}_{\!\!\!\!\!x}\mathop{\int}\nolimits_{\!\!\!\!0}^{{H}_{{\rm{ref}}}}\left(1-\frac{S}{{S}_{{\rm{ref}}}}\right)v\,{\mathrm{d}}z{\mathrm{d}}x,\quad {\mathrm{FW{T}}}_{{\rm{BG}}}={\int}_{\!\!\!\!\!x}\mathop{\int}\nolimits_{\!\!\!\!0}^{{H}_{{\rm{ref}}}}\left({\alpha }_{{\rm{BG}}}-\frac{{D}_{{\rm{BG}}}}{{S}_{{\rm{ref}}}}\right)v\,{\mathrm{d}}z{\mathrm{d}}x,$$

where *D*_BG_ = *α*_BG_⋅*S*_BG_.

### Evaluating the impact of BG freshwater on downstream salinity

The salinity at any downstream grid point can be expressed as the sum of the BG component and the non-BG component:

3 $$S={\alpha }_{{\rm{BG}}}{S}_{{\rm{BG}}}+(1-{\alpha }_{{\rm{BG}}}){S}_{{\rm{nBG}}}.$$

The freshening due to BG freshwater sources is expressed as *δS*_BG_ = *S* − *S*_nBG_. That is, if there is no water sourced from the BG region, *α*_BG_ is zero and *δS*_BG_ is zero, indicating no influence from the BG region. Similarly, if the salinity of BG-sourced water *S*_BG_ is the same as the ambient waters *S*_nBG_, then *δS*_BG_ is also zero. *δS*_BG_ is generally negative, as the contribution from the BG is generally fresher than the contribution from non-BG sources. But where the ambient salinity *S*_nBG_ is generally fresher than *S*_BG_ (e.g., on riverine shelves), *δS*_BG_ is in fact positive (not shown). We use Δ to denote differences between cases (FastRel−FastAcc) and *δ* to denote the impact brought about by the BG-sourced water throughout the paper.

## Supplementary information

Supplementary Information

## Data Availability

The model data are available at 10.5281/zenodo.3967597.

## Code availability

The model source code and the code used to analyze the data are available from the corresponding author on reasonable request.
