# Supplementary material for: Labrador Sea freshening linked to Beaufort Gyre freshwater release
Source: Nat Commun. 2021 Feb 23;12:1229. doi: 10.1038/s41467-021-21470-3 (PMC7902633; doi:10.1038/s41467-021-21470-3)
Supplement: Supplementary file 1 — Supplementary Information [file 41467_2021_21470_MOESM1_ESM.pdf]

Supplementary Information for

Labrador Sea freshening linked to Beaufort Gyre freshwater release

Jiaxu Zhang, Wilbert Weijer, Michael Steele, Wei Cheng, Tarun Verma, and Milena Veneziani

The Supplementary Materials include:

- Supplementary Note 1. Arctic hydrographic conditions in HiLAT03
- Supplementary Note 2. Different treatments of salinity and the salt tracer
- Supplementary Table 1. Transport at four Arctic/Atlantic gateways compared with observations
- Supplementary Table 2. Liquid freshwater transport (FWT) for the fast release and accumulation episodes at each gateway
- Supplementary Figure 1. Positions of the Atlantic/Pacific front in different scenarios
- Supplementary Figure 2. Beaufort Gyre (BG) sourced water at Fram Strait
- Supplementary Figure 3. Understanding the downstream impact
- Supplementary Figure 4. Vertically integrated dye tracer concentration over the upper 200 m
- Supplementary Figure 5. Temporal evolution of standardized salinity anomalies at the exit of theLabrador Sea compared with EN4
- Supplementary Figure 6. Map showing the Arctic Ocean region and the Beaufort Gyre region
- Supplementary Figure 7. Improved Arctic temperature and salinity fields in HiLAT03 compared withthe 1°-resolution version of the model HiLAT10
- Supplementary Figure 8. Arctic freshwater content

Corresponding author: Jiaxu Zhang (jiaxuzh@uw.edu)

Supplementary Note 1. Arctic hydrographic conditions in HiLAT03

The simulated hydrographic conditions of the Arctic Ocean, namely the temperature and salinity structures, are evaluated against the World Ocean Atlas 2013 version 2 (WOA13v2) observational data set[1,2]. Here we define the Arctic Ocean as the ocean area separated by Bering Strait from the Pacific Ocean and Fram Strait, Barents Sea Opening, and Canadian Arctic Archipelago from the Atlantic Ocean (Supplementary Fig. 6). These results are also compared with those of the simulation with a 1° configuration (HiLAT10) of the same model[3] and same forcing conditions. This 1° model is essentially the same as the NCAR ocean-sea-ice model that is used in the Ocean Model Intercomparison Project phase 1 (OMIP1) studies[4,5] except for an updated version of the sea ice model.

Supplementary Fig. 7 shows the Arctic T-S diagram and upper-2000-m temperature and salinity profiles in both simulations compared with the observation. HiLAT03 has a significant improvement of the Arctic thermohaline structure as seen in the T-S diagram, mostly because of more realistic temperature distributions. To be specific, HiLAT03 improves the subsurface (0–100 m) cold biases and especially the mid-to-deep (300–2000 m) warm biases as seen in HiLAT10. The core depth of the Atlantic Water layer is about 600 m in both models, which is deeper than the observed depth of 400 m. But HiLAT03 has an overall fresh bias from 100–2000 m, while the fresh bias is only found in 100–600 m in HiLAT10. We attribute these differences in T-S distributions between HiLAT03 and HiLAT10 to the exclusion of eddy-induced heat and freshwater transport across the North Atlantic polar front associated with the eddy parameterization, which significantly improves the temperature field but slightly biases the salinity field on the downside[6].

Due to the fresh biases discussed here, HiLAT03 has a higher liquid freshwater content (FWC) compared with observations[5,7] (Supplementary Fig. 8a and c). The liquid FWC is defined as

$$FWC = \int_0^{H_{\text{ref}}} \frac{S_{\text{ref}} - S(z)}{S_{\text{ref}}} dz, \tag{1}$$

where the z axis is defined as positive down from the surface $z = 0$; S_{ref} is the reference salinity; H_{ref} is the depth where $S = S_{\text{ref}}$; $S(z)$ is the salinity of the water at depth z. 34.8 is usually used as the reference salinity for the Arctic Ocean since it is the estimated mean Arctic salinity[8]. Due to the fact that the volume-mean salinity of the Arctic Ocean in the model is 34.6 psu, it is reasonable to use 34.6 instead of 34.8 in the calculation. Moreover, the core of the Atlantic Water layer should be excluded from the freshwater bowl (the ocean volume bounded by the surface and the 34.8 isohaline), as indicated in the T-S diagram that the observed temperature maximum falls on the right side of the 34.8 salinity line (Supplementary Fig. 7d). Using 34.6 for the model will prevent the inclusion of a thick Atlantic Water layer into the FWC calculation since the modeled maximum temperature falls on the left of the 34.8 salinity line. Once 34.6 is used, the modeled FWC (Supplementary Fig. 8b) has a similar distribution and magnitude as observed.

Supplementary Note 2. Different treatments of salinity and the salt tracer

We interpret the salt tracer (D_{BG}, or rather, $D_{\text{BG}}/\alpha_{\text{BG}}$) as a measure of Beaufort Gyre (BG) sourced salinity, but note that the comparison with the active salinity tracer is necessarily inexact. Some discrepancies arise from algorithmic choices like the non-linear advection scheme. But the most important discrepancies probably arise from the treatment of the surface boundary conditions. Consistent with common practice[9–11], the model treats freshwater fluxes as virtual salt fluxes, with a fixed global reference salinity S_0 of 34.8 psu. Similarly, the salinity field is forced with unphysical terms like sea surface salinity restoring. It is not obvious how a regionally initialized ‘compound’ tracer

like D_{BG} (conceptually the product of a volume fraction and salinity) should be treated to be fully consistent with this salinity treatment. In our approach, the passive tracers are not subjected to any freshwater exchange with the atmosphere or sea ice. Since we expect this discrepancy to affect the surface layer mostly, we chose to focus on upper-layer (200 m) salinity.

Supplementary Table 1. Transport at four Arctic/Atlantic gateways compared with observations. Comparisons between HiLAT03 and available observations in terms of volume flux (VF) and liquid freshwater flux (FWF) estimated at selected Arctic/Atlantic gateways (see Methods). Periods used for calculation are consistent with observational periods. Numbers shown for observations are means and uncertainties, while numbers shown for the model are means and monthly standard deviations (std). Positive numbers indicate equatorward transports. FWF of the observations is relative to 34.8 psu, except that Tsubouchi et al. (2018)[12] use a time-variant reference salinity of 34.67 ± 0.02 (annual mean ± 1 std). To be consistent with the rest of the paper, we calculate the model FWF relative to 34.6 psu. Note that Jones Sound is not resolved in HiLAT03, so its transport can be considered as part of the Lancaster Sound transport in the model.

		Observation			HiLAT03	
	Period	Study	VF (Sv)	FWF (mSv)	VF (Sv)	FWF (mSv)
Nares Strait	Aug03–Jul06	Münchow et al. (2016)[13]	0.71 ± 0.09	32 ± 5.7	0.69 ± 0.23	21.9 ± 6.3
	Aug07–Jul09	Münchow et al. (2016)[13]	1.03 ± 0.11	54 ± 9.3	0.75 ± 0.20	25.5 ± 5.5
Lancaster Sound	1998–2006	Peterson et al. (2012)[14]	0.53 ± 0.13	32 ± 6	1.05 ± 0.30	77.1 ± 16.7
Jones Sound	1998–2002	Melling et al. (2008)[15]	0.3 ± 0.1	12 ± 4	-	-
Davis Strait	Sep05–Aug06	Tsubouchi et al. (2018)[12]	2.1 ± 0.7	109 ± 13	1.79 ± 0.47	104.9 ± 23.8
	Oct04–Sep09	Curry et al. (2014)[16]	1.6 ± 0.5	93 ± 6	1.72 ± 0.49	88.0 ± 24.6
Fram Strait	Sep05–Aug06	Tsubouchi et al. (2018)[12]	1.1 ± 1.2	79 ± 22	1.97 ± 1.80	67.0 ± 16.2
	1999–2010*	Marnela et al. (2016)[17]	0.8 ± 1.5	66 ± 9	1.68 ± 0.56	52.5 ± 10.3

* Observation covers 8 years during this period: 1999–2002, 2004–05, 2008, 2010, with annual std. HiLAT03 shows 1999–2009 with annual std.

Supplementary Table 2. Liquid freshwater transport (FWT) for the fast release and accumulation episodes at each gateway. Total and Beaufort Gyre (BG) sourced liquid FWT (see Methods) estimated at four Arctic/Atlantic gateways. Numbers listed in this table correspond to the bar plot of Fig. 3c. Climatology is calculated for the entire forcing cycle, namely 1948–2009.

	Total liquid FWT (mSv)				BG-sourced FWT (mSv)			BG ratio (%)		
	Climatology	FastRel	FastAcc	Diff	FastRel	FastAcc	Diff	FastRel	FastAcc	Diff
Nares Strait	27.8	32.3	24.9	7.4	19.1	10.3	8.9	61.7	41.3	20.4
Lancaster Sound	74.7	84.8	69.1	15.6	58.0	37.7	20.4	69.5	54.9	14.5
Davis Strait	96.9	111.9	89.0	22.8	70.2	45.2	25.0	63.3	52.5	10.9
Fram Strait	67.7	83.5	62.8	20.6	30.3	19.3	10.9	38.2	31.8	6.5

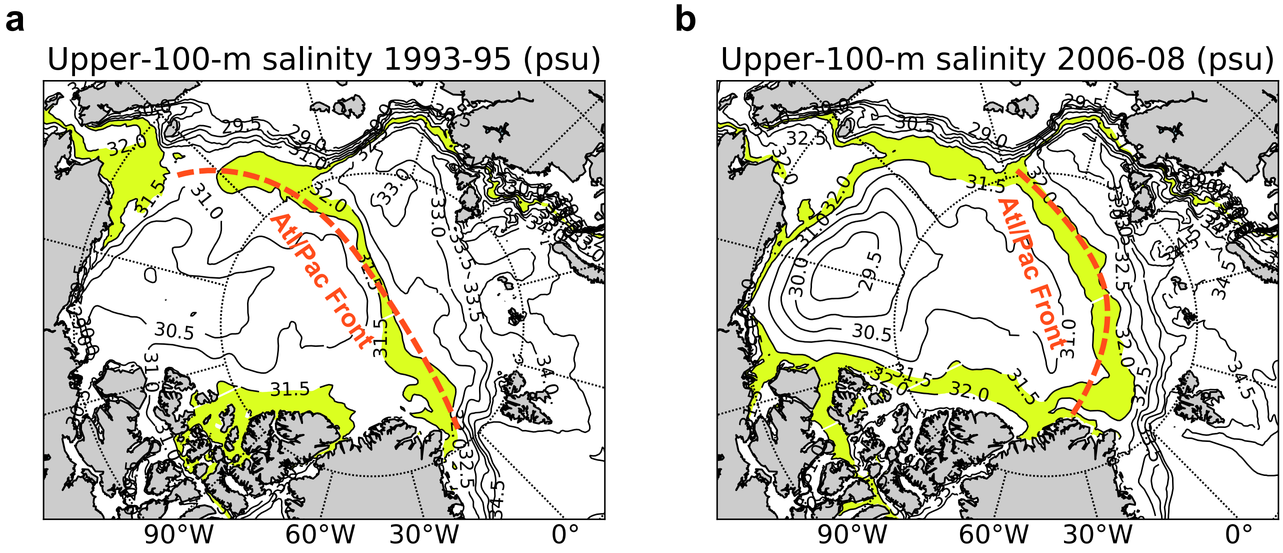

Supplementary Figure 1. Positions of the Atlantic/Pacific front in different scenarios. Arctic salinity for the last three years of **a** FastRel (1993–95) and **b** FastAcc (2006–08) averaged over the upper 100 m depths. The band between 31.5 and 32.0 psu are highlighted. The red dashes illustrate the position of the Atlantic/Pacific front in each scenario as implied from the salinity distributions, since the Pacific water is in general fresher than the Atlantic water.

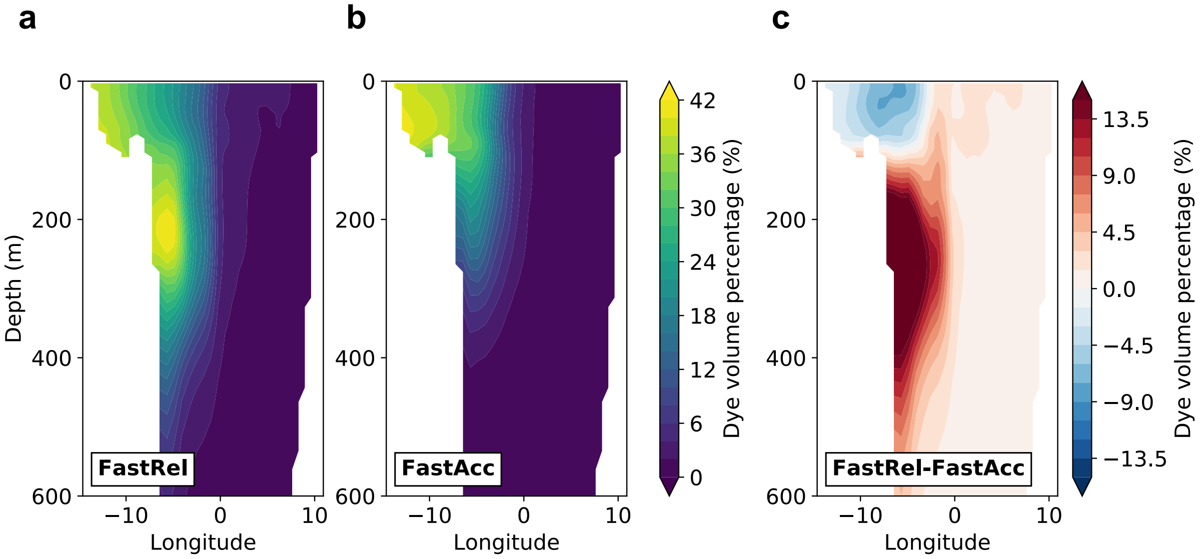

Supplementary Figure 2. Beaufort Gyre (BG) sourced water at Fram Strait. Vertical distribution of the dye tracer, as an indication of BG-water volume fraction, at Fram Strait averaged over the last 3 years of the **a** FastRel episode, **b** FastAcc episode, and **c** the difference between them. Although the overall BG volume transport is higher in FastRel, the BG-sourced water is in deeper depths, leaving the shallow coastal areas less impacted by BG water compared with the FastAcc case.

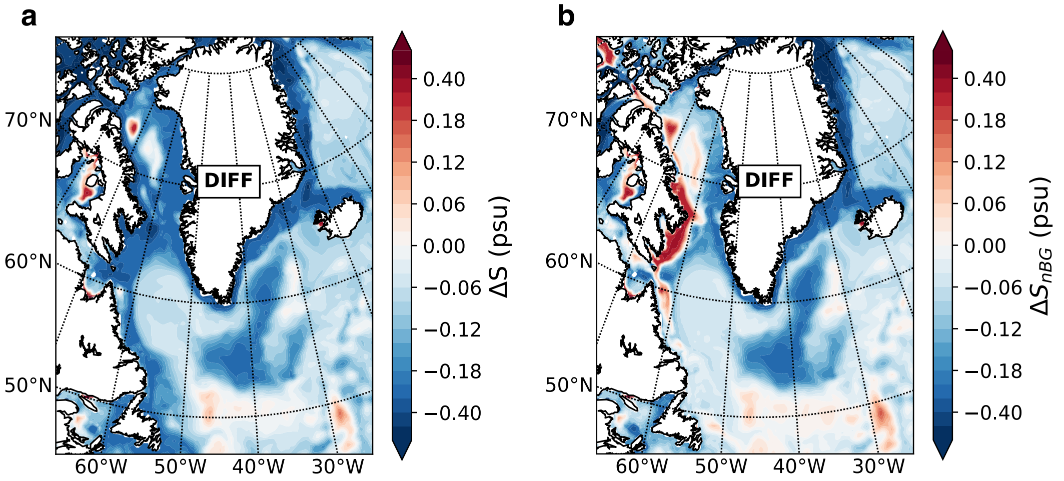

Supplementary Figure 3. Understanding the downstream impact. Similar as Fig. 4c, but for differences of **a** salinity (ΔS) and **b** diagnosed impact from non-Beaufort Gyre (BG) sources (ΔS_{nBG}). See Methods for the definition of S_{nBG}. The difference between **a** and **b** gives the estimated impact from BG-sourced water as shown in Fig. 4c, that is, $\Delta\delta S_{\mathrm{BG}} = \Delta S - \Delta S_{\mathrm{nBG}}$. The upper ocean salinity during 1993–95 is generally fresher than during 2006–08, especially over coastal areas. The freshening at Nares Strait and the northern East Greenland Current is predominantly caused by fresher Pacific waters (not from the BG).

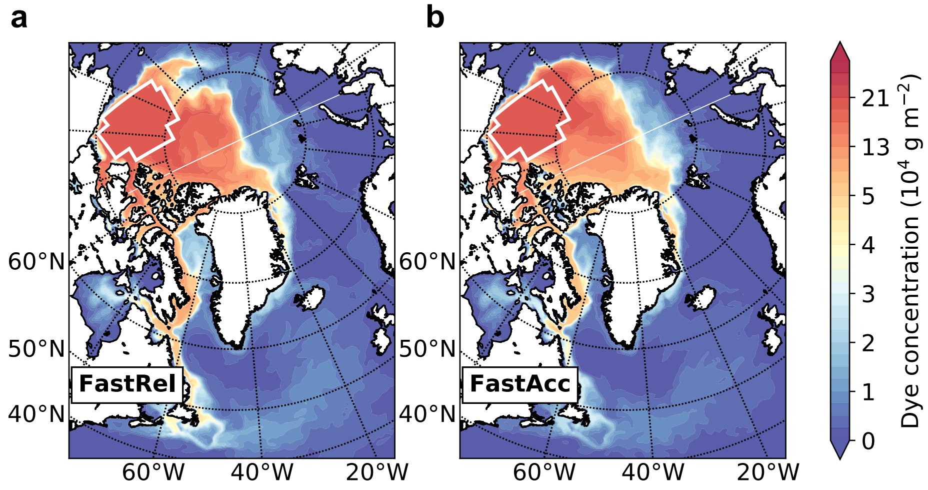

Supplementary Figure 4. Vertically integrated dye tracer concentration over the upper 200 m. Same as Fig. 2a and b, but for the upper 200 m only. Low dye concentration is found in the Labrador Sea interior, which is different from Fig. 2, indicating dye tracer is mostly transported to depths deeper than 200 m.

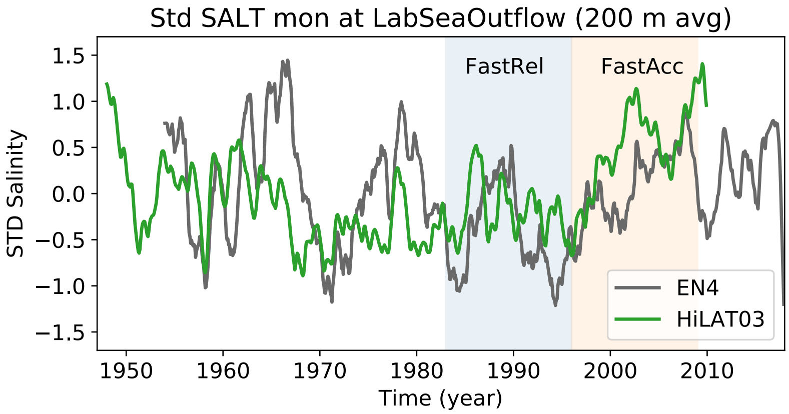

Supplementary Figure 5. Temporal evolution of standardized salinity anomalies at the exit of the Labrador Sea compared with EN4. Salinity is averaged over 49–54°N, 50–56°W (indicated by the green box in Fig. 1a) for the upper 200 m in both model and EN4. This location is chosen because of a better observational coverage in EN4 before 2000s than in the rest of the western Labrador Sea. Model (green) and EN4 (black) monthly data are first standardized and then smoothed with a 13-point unweighted running average window. EN4 version 4.2.1 is an objectively analysed subsurface temperature and salinity data set (https://www.metoffice.gov.uk/hadobs/en4/).

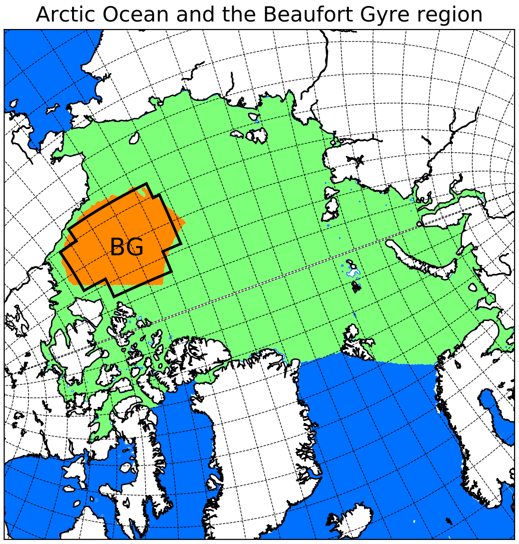

Supplementary Figure 6. Map showing the Arctic Ocean region and the Beaufort Gyre region. The Arctic Ocean region is shown by the green area (including the orange area). The way that the BG region is defined in this study (black zigzag box) is to maximize its overlap with the traditional definition[18] (70.5°N to 80.5°N and 130°W to 170°W with water depth greater than 300 m; orange area) while keeping the boundaries aligned with the model grid (dashed lines in increments of 20 grid spacings in both directions).

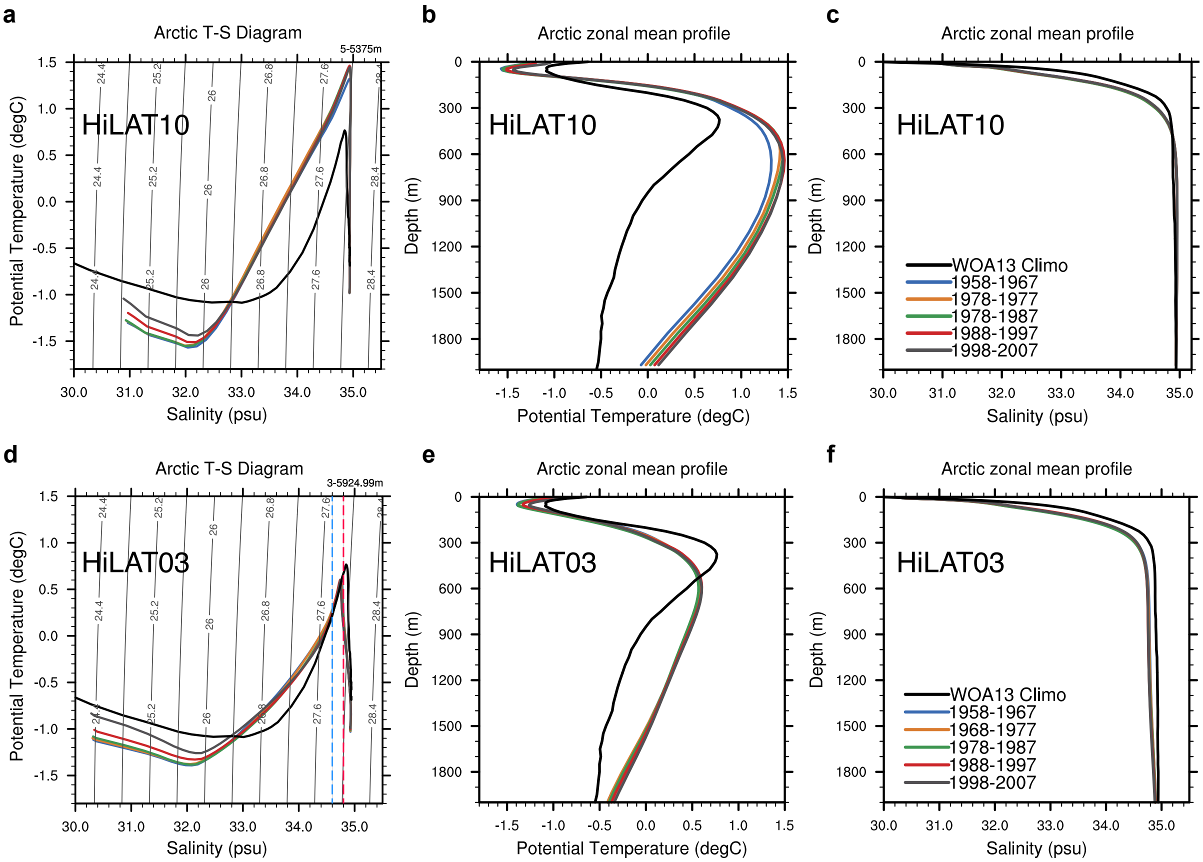

Supplementary Figure 7. Improved Arctic temperature and salinity fields in HiLAT03 compared with the 1°-resolution version of the model HiLAT10. The Arctic **a** T-S diagram, **b** temperature profile, and **c** salinity profile of different decades in HiLAT10, which is a low-resolution configuration of the model used in this study. Black curves are the WOA13v2 1955–2012 climatology[1,2]. **d**–**f** Same as **a**–**c** but for the HiLAT03 configuration, which is used in the current study. Red and blue dashed lines in **d** indicates salinities of 34.8 psu (traditional Arctic reference salinity) and 34.6 psu (reference salinity used in this study), respectively.

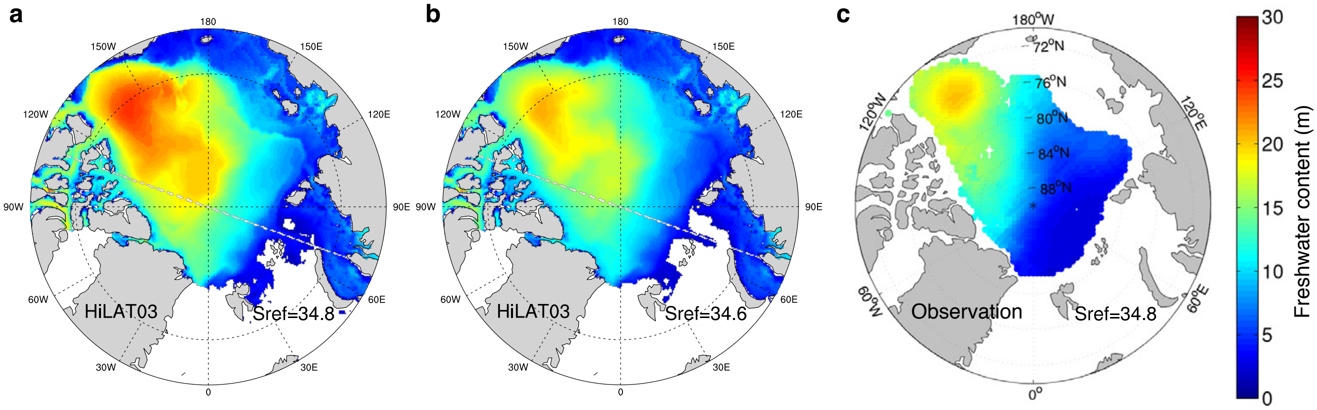

Supplementary Figure 8. Arctic freshwater content. Model simulated liquid freshwater content of the 1993–2002 period relative to **a** 34.8 psu and **b** 34.6 psu are compared with **c** the observational data set[5,7] relative to 34.8 psu. The vertical integration is taken from surface to the depth where salinity is equal to the reference salinity.

Supplementary References

1. Locarnini, R. A. *et al.* World Ocean Atlas 2013, Volume 1: Temperature. *NOAA Atlas NESDIS 73, NOAA/NESDIS, U.S. Dept. Commer. Washington, D.C.* (2013).
2. Zweng, M. M. *et al.* World Ocean Atlas 2013, Volume 2: Salinity. *NOAA Atlas NESDIS 73, NOAA/NESDIS, U.S. Dept. Commer. Washington, D.C.* (2013).
3. Hecht, M. *et al.* E3SMv0-HiLAT: A modified climate system model targeted for the study of high-latitude processes. *J. Adv. Model. Earth Syst.* **11**, 2814–2843, DOI: 10.1029/2018MS001524 (2019).
4. Danabasoglu, G. *et al.* North Atlantic simulations in Coordinated Ocean-ice Reference Experiments phase II (CORE-II). Part I: Mean states. *Ocean. Model.* **73**, 76–107, DOI: 10.1016/j.ocemod.2013.10.005 (2014).
5. Wang, Q. *et al.* An assessment of the Arctic Ocean in a suite of interannual CORE-II simulations. Part II: Liquid freshwater. *Ocean. Model.* **99**, 86–109, DOI: 10.1016/j.ocemod.2015.12.009 (2016).
6. von Storch, J.-S., Haak, H., Hertwig, E. & Fast, I. Vertical heat and salt fluxes due to resolved and parameterized meso-scale Eddies. *Ocean. Model.* **108**, 1–19, DOI: 10.1016/j.ocemod.2016.10.001 (2016).
7. Rabe, B. *et al.* Arctic Ocean basin liquid freshwater storage trend 1992-2012. *Geophys. Res. Lett.* **41**, 961–968, DOI: 10.1002/2013GL058121 (2014).
8. Aagaard, K. & Carmack, E. C. The role of sea ice and other fresh water in the Arctic circulation. *J. Geophys. Res.* **94**, 14485–14498, DOI: 10.1029/JC094iC10p14485 (1989).
9. Dukhovskoy, D. S. *et al.* Greenland freshwater pathways in the sub-Arctic Seas from model experiments with passive tracers. *J. Geophys. Res. Ocean.* **121**, 877–907, DOI: 10.1002/2015JC011290 (2016).
10. Luo, H. *et al.* Oceanic transport of surface meltwater from the southern Greenland ice sheet. *Nat. Geosci.* **9**, 528–532, DOI: 10.1038/ngeo2708 (2016).
11. Böning, C. W., Behrens, E., Biastoch, A., Getzlaff, K. & Bamber, J. L. Emerging impact of Greenland meltwater on deepwater formation in the North Atlantic Ocean. *Nat. Geosci.* **9**, 523–527, DOI: 10.1038/ngeo2740 (2016).
12. Tsubouchi, T. *et al.* The Arctic Ocean seasonal cycles of heat and freshwater fluxes: Observation-based inverse estimates. *J. Phys. Ocean.* **48**, 2029–2055, DOI: 10.1175/JPO-D-17-0239.1 (2018).
13. Münchow, A. Volume and Freshwater Flux Observations from Nares Strait to the West of Greenland at Daily Time Scales from 2003 to 2009. *J. Phys. Ocean.* **46**, 141–157, DOI: 10.1175/JPO-D-15-0093.1 (2016).
14. Peterson, I., Hamilton, J., Prinsenberg, S. & Pettipas, R. Wind-forcing of volume transport through Lancaster Sound. *J. Geophys. Res. Ocean.* **117**, C11018, DOI: 10.1029/2012JC008140 (2012).
15. Melling, H. *et al.* Fresh-water fluxes via Pacific and Arctic outflows across the Canadian polar shelf. In *Arctic–Subarctic Ocean Fluxes*, 193–247, DOI: 10.1007/978-1-4020-6774-7_10 (Springer Netherlands, Dordrecht, 2008).
16. Curry, B., Lee, C. M., Petrie, B., Moritz, R. E. & Kwok, R. Multiyear Volume, Liquid Freshwater, and Sea Ice Transports through Davis Strait, 2004–10*. *J. Phys. Ocean.* **44**, 1244–1266, DOI: 10.1175/JPO-D-13-0177.1 (2014).

17. Marnela, M., Rudels, B., Goszczko, I., Beszczynska-Möller, A. & Schauer, U. Fram Strait and Greenland Sea transports, water masses, and water mass transformations 1999–2010 (and beyond). *J. Geophys. Res. Ocean.* **121**, 2314–2346, DOI: 10.1002/2015JC011312 (2016).

18. Proshutinsky, A. *et al.* Beaufort Gyre freshwater reservoir: State and variability from observations. *J. Geophys. Res.* **114**, C00A10, DOI: 10.1029/2008JC005104 (2009).
